# Influence of Poly(Ethylene Glycol) Dimethacrylates’ Chain Length on Electrical Conductivity and Other Selected Physicochemical Properties of Thermally Sensitive N-isopropylacrylamide Derivatives

**DOI:** 10.3390/polym16192786

**Published:** 2024-09-30

**Authors:** Agnieszka Gola, Borys Podżus, Kinga Gruszka, Witold Musiał

**Affiliations:** Department of Physical Chemistry and Biophysics, Pharmaceutical Faculty, Wroclaw Medical University, Borowska 211, 50-556 Wroclaw, Poland; agnieszka.gola@umw.edu.pl (A.G.); borys.podzus@gmail.com (B.P.); kinga.gruszka@poczta.onet.pl (K.G.)

**Keywords:** nanoparticles, N-isopropylacrylamide, poly(ethylene glycol) dimethacrylates, lower critical temperature solution, anionic initiator, ammonium persulfate, electrical conductivity

## Abstract

Thermosensitive polymers P1–P6 of N-isopropylacrylamide (PNIPA) and poly(ethylene glycol) dimethacrylates (PEGDMAs), av. Mn 550–20,000, were synthesized via surfactant-free precipitation polymerization (SFPP) using ammonium persulfate (APS) at 70 °C. The polymerization course was monitored by the conductivity. The hydrodynamic diameters (HDs) and the polydispersity indexes (PDIs) of the aqueous dispersion of P1–P6 in the 18–45 °C range, assessed via dynamic light scattering (DLS), were at 18° as follows (nm): 73.95 ± 19.51 (PDI 0.57 ± 0.08), 74.62 ± 0.76 (PDI 0.56 ± 0,01), 69.45 ± 1.47 (PDI 0.57 ± 0.03), 196.2 ± 2.50 (PDI 0.53 ± 0.04), 194.30 ± 3.36 (PDI 0.56 ± 0.04), 81.99 ± 0.53 (PDI 0.56 ± 0.01), 76.87 ± 0.30 (PDI 0.54 ± 0.01), respectively. The electrophoretic mobilities estimated the zeta potential (ZP) in the 18–45 °C range, and at 18 °C they were as follows (mV): −2.57 ± 0.10, −4.32 ± 0.67, −5.34 ± 0.95, −-3.02 ± 0.76, −4.71 ± 2.69, −2.30 ± 0.36, −2.86 ± 0.42 for polymer dispersion P1–P6. The polymers were characterized by attenuated total reflectance–Fourier transform infrared spectroscopy (ATR-FTIR), H nuclear magnetic resonance (^1^H NMR), thermogravimetric analysis (TG/DTA), Differential Scanning Calorimetry (DSC), and powder X-ray diffraction analysis (PXRD). The length of the cross-linker chain influences the physicochemical properties of the obtained polymers.

## 1. Introduction

The development of nanotechnology in pharmaceutical and biomedical sciences is the most promising area of research in the context of improving the effectiveness of pharmacotherapy. The main requirements of modern pharmacological treatment are to improve its specificity by developing targeted therapy, to adjust the optimal pharmacokinetics, to reduce adverse reactions, and to avoid the drug substance’s impact on healthy tissues. These features are particularly beneficial in the treatment of antibiotics, anticancer, and antiviral drugs [1].

Potential materials meeting the requirements of modern pharmacotherapy are three-dimensional polymer networks that react to environmental stimuli in a reversible manner, e.g., anionic or cationic polymers or ion-sensitive polymers, which may reversibly enable release of a drug from their structure, depending on pH or specific ion presence [2,3,4]. Due to their specific responsive nature to external stimuli, these materials are also commonly referred to as smart polymers. The variety of possible modifications to their structure, which impacts their physicochemical properties, supports their utility as a drug carrier. Properly selected features of such material could enhance their practical use in the medical sector [5,6,7]. The synthesis of polymers with cross-linking agents allows the production of products possessing different reaction thresholds towards external factors, like temperature, pH, and ionic strength, in contrast to the basic linear structure of the polymer. Currently, the objective is to procure polymers with precisely selected parameters that would enable the overcoming of anatomical barriers and the controlled release of the drug substance at the intended site [8,9].

Sensitivity to external stimuli is a characteristic commonly displayed by various biopolymers found in living organisms. The observation of these polymers interacting with their environment led to the proposition of manufacturing synthetic analogues with regulated sensitivity. Resilin is an instance of such a compound, a protein present in the cuticle of the fruit fly (*Drosophila melanogaster*), characterized by above-average mechanical strength and elasticity. The features that define resilin become apparent upon exposure to mechanical stimuli, and these changes are reversible. Resilin stores energy during periods of stretching and then rapidly releases this energy, thereby enabling dynamic movements. Following each cycle of stretching and contraction, the material returns to its original form without any loss of functionality [10,11,12,13]. In response to stressful conditions, both prokaryotic and eukaryotic organisms have evolved a protective mechanism comprising guardian proteins, also known as heat shock proteins (HSPs). These proteins are responsible for maintaining cellular homeostasis and are induced by a variety of stressors, including heat shock. Upon the removal of the stressor, HSPs can return to their normal state [14,15]

Polymers that respond to changes in temperature, particularly those with a phase transition temperature near the human body’s physiological temperature, are regarded as an effective drug delivery system with controlled release of active substances [16]. The applicability of temperature-responsive polymers as carriers for selected active pharmaceutical agents was investigated. Poly N-isopropylacrylamide (PNIPA) was studied as a mesalazine carrier [17], whereas poly(N-isopropylacrylamide-co-acrylic acid) was applied in research on doxorubicin [18] and ibuprofen [19]. Voriconazole was investigated using chitosan-graft-poly(N-isopropyl acrylamide) and polyvinyl alcohol [20]. The ease of adjusting the temperature stimulus intensity in experimental settings and the feasibility of conducting in vitro and in vivo testing account for the widespread usage of thermally responsive polymers in research [16]. These polymers are also anticipated for application in regenerative medicine to retrieve cells of two-dimensional tissues from culture media [21].

Poly PNIPA is a temperature-responsive polymer that has gained attention from medical researchers due to its phase transition temperature, which is similar to the physiological temperature of the human body [22]. At its lower critical solution temperature (LCST) of 32 °C, according to our measurements, PNIPA may undergo a rapid phase transition. Below this temperature, PNIPA mixes with water in an unlimited way due to the formation of hydrogen bonds between the hydrophilic groups (-CONH-) and the solvent molecules. This results in the solvation of the polymer [22]. The binding of water molecules by hydrophilic fragments of the PNIPA molecule leads to a decrease in the strength of polymer–polymer interactions and the loosening of the macromolecule chain structure, which takes on the so-called coil form [23]. As the ambient temperature increases, the hydrogen bonds weaken, while the strength of hydrophobic interactions between isopropyl substituents (-CH(CH_3_)_2_) [22] increases. This, along with the increase in the entropy of water molecules at isopropyl groups, leads to the release of water from connections with amide groups. Upon reaching and surpassing the LCST, the chain conformation transforms into a hydrophobic globular form (globule), causing polymer aggregates to precipitate from the solution [24]. PNIPA and its derivatives are potential carriers for drug substances in targeted therapy and the development of new controlled drug release systems due to numerous studies published in recent decades [25,26,27,28,29,30]. The significant advantage of using this polymer in these applications is its lack of cytotoxicity [31,32]. The modification of the linear structure of PNIPA during the synthesis of hydrogels based on it achieved biocompatibility and biodegradability [33,34]. The combination of PNIPA with cross-linking agents creates better conditions for loading the drug into the hydrogel network and enables drug release after reaching an LCST equal to or higher than the desired temperature [21].

The development of controlled drug release systems involves the linking of PNIPA chains with biodegradable compounds, such as poly(ε-caprolactone) dimethacrylate (PCLDMA) and bisacryloylcystamine (BACy), which are easily degraded at the site of application [35,36]. This construction allows for a rapid release of the initial dose of the drug substance after the biodegradable components degrade. The maintenance dose remaining in the polymer network is released gradually due to the gradual pushing out of the drug molecules from the shrunken PNIPA chains [33].

An important aspect of drug carriers in targeted anticancer therapy is the controlled release of cytostatic substances. This is necessary to prevent the drug from being released into the bloodstream and causing systemic effects. A beneficial carrier should provide specific drug permeability and drug targeting to enable drug activity in desirable regions of the body, e.g., in the cancerous tissue or in infected body areas, and should not demonstrate toxic effects on healthy tissues [35,36,37,38,39]. The use of PNIPA as a carrier in targeted therapy is related to its thermosensitive properties and the processes in cancer cells. Cancer tissue’s rapidly dividing cells require a constant supply of nutrients, which is the responsibility of the blood vessels produced during cancer angiogenesis.

One feature of vessels created in this manner is the absence of smooth muscles, which renders them unresponsive to neurotransmitter-induced contractions. As a result, the blood flow through the vessels remains constant, leading to an increase in temperature in the neoplastic region [40]. An appropriately modified structure of the PNIPA hydrogel, with an LCST equal to the temperature of the tumor tissue (1–2 °C higher than the physiological temperature), could be used as a carrier for cytostatic drugs.

The use of PNIPA in cell culture for regenerative medicine is based on changes in surface polarity and hydration, which affect cell adsorption. Culturing is performed in a medium with a temperature above the LCST. This exposes isopropyl groups and eliminates bound water, facilitating cell adhesion. Material recovery is possible by reducing the temperature below 32 °C, which causes hydration and changes in the polymer’s conformation. This results in reduced cell adhesion and detachment of the tissue layer. The method enables the acquisition of cell monolayers from two-dimensional tissues, such as epithelial tissue, without harming the cells or their connections. This is in contrast to other invasive methods, such as mechanical or enzymatic methods [21].

Another medical application for PNIPA is the development of dressings that incorporate chains of the polymer into their structure. The intended use of such materials is the treatment of wounds that are difficult to heal, deep, leaky, and prone to infection, such as burns or surgical incisions. Hybrid hydrogels containing PNIPA in their structure can meet the following requirements: good air permeability, ability to absorb exudate, ensuring proper hydration, and protection against infection. In addition, the transparency of PNIPA-based materials allows the wound healing process to be monitored. On the other hand, the synthesis of hybrid hydrogels allows their properties to be adapted to the needs of the target material [41]. Combinations of PNIPA with microcrystalline cellulose increase the strength of the product [42]: with calcium alginate, they allow the maintenance of appropriate wound moisture [43]; with silver nanoparticles, they provide antibacterial properties [44]; and with boron nitride nanolayers, they improve adhesive properties [45].

Poly(ethylene glycol) dimethacrylates (PEGDMAs) belong to the group of polyoxyethylene glycol derivatives. The presence of double bonds between the carbon atoms of the methacrylic group demonstrates their ability to attach molecules with unpaired electrons, accompanied by the transfer of the radical center [46]. They are most commonly used as cross-linkers in the synthesis of polymer hydrogels and branched polymers by radical polymerization [47,48]. In medicine, TETGDMA is used as a component of light-curing dental composites used in aesthetic enamel reconstruction procedures—it is a component of the organic phase formed by methacrylate resins. It is also considered as a replacement for the components of resin matrices currently used in medicine.

The aim of this work was to synthesize the cross-linked structure of six PNIPA derivatives and to determine the effect of PEGDMAs of different chain lengths, used in the synthesis as cross-linking agents, on the physicochemical properties of the PNIPA derivatives obtained. Analysis of the results of the instrumental tests carried out made it possible to determine the usefulness of the product synthesized as a potential temperature-triggered drug carrier.

## 2. Materials and Methods

### 2.1. Materials

N-isopropylacrylamide (NIPA) (99% St. Louis, MO, USA), ammonium persulfate (APS) (98%, Sternheim, Germany), poly(ethylene glycol) dimethacrylate (PEGDMA) (average Mn ~550, ~750, ~2000, ~6000, ~10000, ~20000, St. Louis, MO, USA), were purchased from Sigma Aldrich. Dialysis tubing cellulose membrane (MWCO 12,000–14,000 Da St. Louis, MO, USA) was also obtained from Sigma Aldrich. The deionized water (<0.06 μS cm^−1^) was filtered using an HLP 20 system (microfiltration capsule 0.22 μm, Hydrolab, Straszyn, Poland). It met the requirements of the PN-EN ISO 3696:1999 standards for analytical laboratories. All chemicals and solvents were used as received without further purification or modification.

### 2.2. Synthesis

Six thermosensitive homopolymers (P1–P6) were synthesized using cross-linking agent PEGDMA and anionic initiator APS via free radical precipitation polymerization without the addition of a surfactant (SFPP). The reaction was conducted for 6 h at 70 °C in an aqueous environment with continuous stirring (2500 rpm) under an inert N_2_ atmosphere. Table 1 shows the substrate composition and product names and acronyms.

The post-reaction mixture underwent purification through forced equilibrium dialysis (FED) against deionized water, followed by lyophilization for 26 h using Alpha 1-2 LD (Martin Christ Freeze Dryers, Osterode am Harz, Germany). The resulting polymers were analyzed using ATR-FTIR, TG, DSC, and PXRD techniques.

### 2.3. Conductivity Analysis

The CC-505 conductometer (Elmetron, Gliwice, Poland) was used to measure the conductivity of the reaction mixture during polymerization at a constant temperature of 70 °C. The conductivity was also measured after synthesis during the cooling process. The instrument had an accuracy of up to 19,999 mS·cm^−1^ ± 0.1% for values below 20,000 mS·cm^−1^ and ± 0.25% for values above 20,000 mS·cm^−1^. The conductometer was fitted with an EC-60 immersion conductometric sensor featuring platinum electrodes and a glass housing (K = 1.0 ± 0.2 cm^−1^, Elmetron, Gliwice, Poland), as well as a Pt-1000A temperature sensor (0–100 ± 0.35 °C). Both sensors were continuously submerged in the reaction mixture. Temperature compensation was manually ensured during the polymerization reaction and automatically during the cooling process.

### 2.4. Attenuated Total Reflection Fourier Transform Infrared Spectroscopy Measurements

The Nicolet iS50 FT-IR spectrometer, equipped with a monolithic diamond crystal universal ATR sampling accessory (Thermo Fisher Scientific, Madison, WI, USA), was used to conduct attenuated total reflection Fourier transform infrared analysis (ATR-FTIR). The prepared samples P1–P6 were analyzed in the infrared region from 4000 to 400 cm^−1^ with an average of 32 scans taken at resolution 4 cm^−1^ ± 0.01 cm^−1^ at room temperature. A deuterated L-alanine-doped triglycene sulphate detector (DLaTGS) was used. The ATR module was cleaned with methanol and dried before measuring the sample. The background spectrum was obtained by using a blank ATR crystal and automatically subtracted from the sample spectrum. The ATR-FTIR spectra of substrates in commercial form and the lyophilized polymerization products were measured under identical instrument conditions at ambient temperature. The spectral data were processed using OMNIC software (version 9, Thermo Fisher Scientific, Madison, WI, USA), and the substances were compared by qualitative analysis.

### 2.5. Hydrodynamic Diameter and Polydispersity Index Measurements

The dynamic light scattering (DLS) technique was used to measure the hydrodynamic diameter (HD), distributions, and polydispersity index (PDI) of the dispersion of aqueous polymer particles. A Zetasizer Nano ZS ZEN3600 device (Malvern Instruments, Malvern, UK) equipped with a standard red He-Ne laser (4 mW, λ = 633 nm) was used for the measurements. A sensitive avalanche photodiode detector (APD) was placed at a 173° angle, and non-invasive backscattering (NIBS) technology was applied. A laser beam attenuator was used to regulate the light intensity during the measurement. The measurements were conducted in a translucent polyacrylic disposable DTS-0012 cuvette (Malvern Instruments, Malvern, UK) filled with 1 mL of purified polymer dispersion that was not diluted and had no precipitation after dialysis. The cuvette was placed in a temperature-controlled measurement cell. The sample was equilibrated for 240 s before measurement at each new temperature. DLS measurements were recorded in 1 °C increments from 18 to 45 °C. The number of runs per measurement was automatically adjusted within the range of 10–100. The cumulants analysis algorithm was used to estimate the HD and PDI parameters. The methods for calculating these parameters are defined in ISO standard documents: ISO 13321:1996E and ISO 22412:2008 [49,50,51]. The refractive index and viscosity of water were used as dispersant parameters, while polystyrene latex was used as the material parameter for calculations. The average values of HD and PDI data were obtained from five consecutive measurements at each temperature, as indicated in the figures. The repeated results were in good agreement. The size distribution was presented by intensity, with PDI value 0 for highly monodispersed standard. Zetasizer^®^ software version 7.10 was used to create custom standard operating protocols (SOPs) for use on subsequent samples without modification and to process data from DLS measurements. The statistical analysis was conducted using descriptive statistical methods.

### 2.6. Zeta Potential Measurements

A Zetasizer Nano ZS ZEN3600 device (Malvern Instruments, Malvern, UK) was used to measure the zeta potential of polymer particles in aqueous dispersion. The measurement was carried out using the laser Doppler electrophoresis technique (laser Doppler velocimetry, LDV). The Henry equation was approximated using the Smoluchowski model (f(Ka) = 1.5). Measurements were taken using a 0.75 mL U-shaped plastic capillary cuvette with built-in gold-plated copper electrodes (Malvern Instruments, Malvern, UK). The temperature range was 18–45 °C, with measurements taken every one degree and an equilibration time of 120 s at each temperature. The zeta potential value was determined by averaging five measurements at each temperature. The measurements were recorded with Zetasizer^®^ software (version 7.11). The statistical analysis was conducted using descriptive statistical methods.

### 2.7. Thermogravimetric Measurements

A TG 209 F1 Libra instrument with an automatic sample changer (ASC) (Erich NETZSCH GmbH and Co. Holding KG, Selb, Germany) was used to conduct thermogravimetric analysis (TGA). The thermal decomposition experiment was carried out under non-isothermal heating conditions. The samples weighing 5.0 ± 0.02 mg were heated from 25 to 800 °C at heating rate 5.0 °C·min^−1^ under high-purity nitrogen atmosphere with a flow rate of 50 mL·min^−1^. Standard, opened alumina, Al_2_O_3_, crucibles (150 µL) were used. The lyophilized material was compacted into the crucible with a rammer. The material was not graded. Weight loss measurements were recorded continuously, as a function of temperature and time. The data were recorded and processed using Netzsch Proteus 7.1.0 analysis software (Selb, Germany).

### 2.8. Differential Scanning Calorimetry Measurements

A DSC 214 Polyma instrument (Netzsch, Selb, Germany) equipped with an Intracooler IC70 (Netzsch, Selb, Germany) was used to perform the differential scanning calorimetry. The lyophilized samples, weighing3.0 ± 0.07 mg, were placed in standard aluminum crucibles (~25 μL) with vented covers and scanned against an empty reference crucible of the same type. All measurements were conducted under high-purity nitrogen atmosphere at a flow rate of 50 mL·min^−1^. The samples underwent programmed heating and cooling between 25 and 240 °C at a rate of 5.0°C·min^−1^. The experimental running conditions, according to set temperature program heating/cooling/heating/cooling/heating, were as follows: heated to 240°C, isothermal 2 min, cooled to 25°C, isothermal 5 min. The experimental data were analyzed using Netzsch Proteus^®^ 7.1.0 analysis software (Netzsch, Selb, Germany). The synthesized polymers P1–P6 were analyzed using differential scanning calorimetry to determine the characteristic quantities of the glass transition Tg. These quantities include the onset, midpoint, inflection, endset temperature, and glass transition height ΔCp.

### 2.9. Powder X-ray Diffraction Measurements

The P1–P6 polymer samples were ground in an agate mortar and analyzed using a Bruker D2 PHASER X-ray diffractometer (Bruker AXS, Karlsruhe, Germany) equipped with a LynxEYE detector. The X-ray powder diffraction patterns were recorded in the Bragg–Brentano (θ/2θ) horizontal geometry. For XRD measurements, the samples were irradiated between 5° and 70° with 0.02° increments, using monochromatic Ni-filtered CuKα_1.2_ radiation (λ = 1.5418 Å) at an angle of 2θ and τ = 4 s/step. The voltage was set to 30 kV and the electric current to 10 mA. The rotation of sample was 15 min^−1^, the divergence slit 1.0 mm, and the shutter 0.5 mm. A sample holder with a small cavity of 20 mm × 0.5 mm and a diameter of 51.5 mm (Brucker AXS, C79298-A3244-B261, Karlsruhe, Germany) was used to measure the samples at 295 K in ambient atmosphere. The samples were placed on the holder and pressed to create a flat surface.

Diffrac.Eva V 3.2 software (Bruker AXS, Karlsruhe, Germany) was used to analyze and process the PXRD data.

## 3. Results

### 3.1. Synthesis

Polymers P1–P6 were synthesized via a free radical polymerization technique according to Pelton’s methodology described in numerous articles.

**Figure 1:**
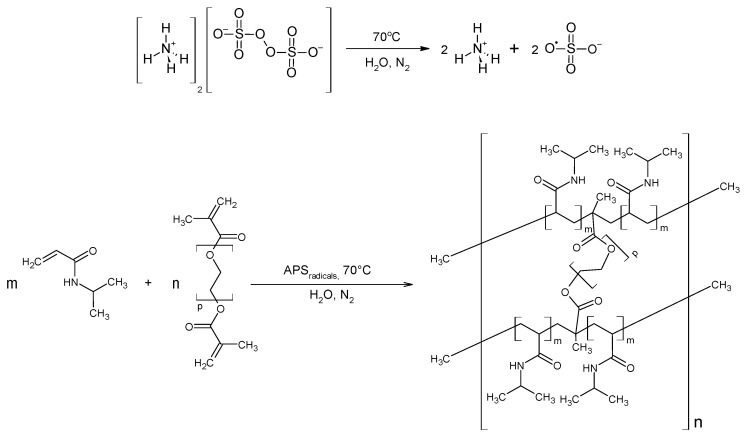
Polymerization reaction scheme of NIPA with PEGDMA under the experimental conditions employed in this study, together with the suggested polymer structure.

The synthesis process for polymers P1–P6 is displayed in a chosen exemplary general scheme, as illustrated in Figure 1.

The molar ratios of NIPA, APS, and cross-linker PEGDMA required for the synthesis of polymers P1–P6 were as follows: NIPA:APS:PEGDMA (Mn~550)—1:0.05:0.02; NIPA:APS:PEGDMA (Mn~750)—1:0.05:0.02; NIPA:APS:PEGDMA (Mn~2000)—1:0.05:0.006; NIPA:APS:PEGDMA (Mn~6000)—1:0.05:0.002;—NIPA:APS:PEGDMA (Mn~10000)—1:0.05:0.001; NIPA:APS:PEGDMA (Mn~20000)—1:0.05:0.0007. The detailed composition of the polymers is given in Table 1.

More information on the synthesis procedure can be found in Section 2.2. From 100 mL of purified polymer solutions, 0.37605 g of P1, 0.37858 g of P2, 0.41609 g of P3, 0.47413 g of P4, 0.46013 g of P5, and 0.43545 g of P6 polymers were obtained through freeze-drying. The resulting products were a white solid with a wattle-like consistency.

### 3.2. Conductivity Measurements

**Figure 2:**
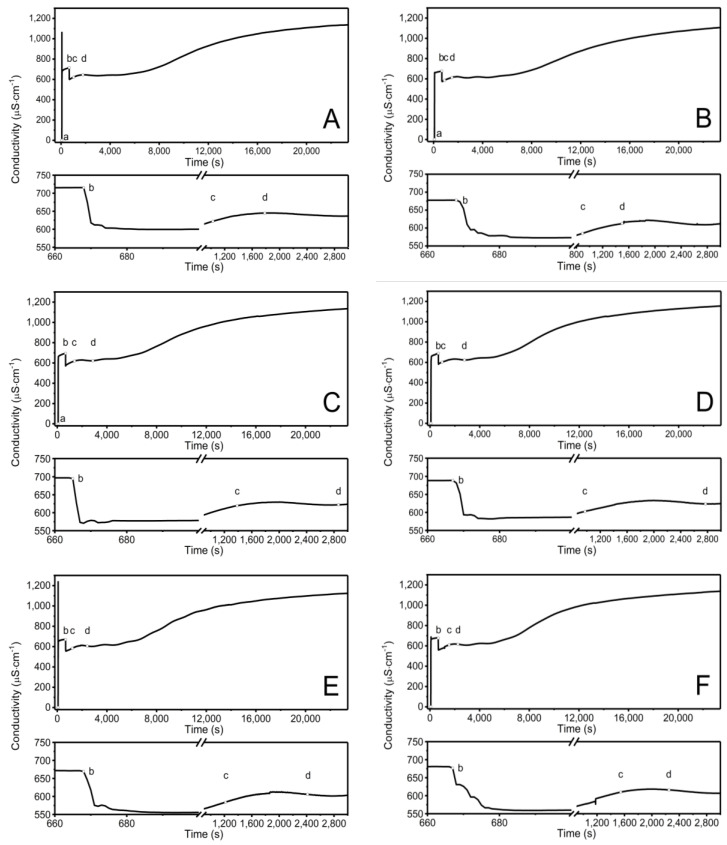
Conductivity changes observed in the reaction systems in the course of synthesis of P1 (**A**), P2 (**B**), P3 (**C**), P4 (**D**), P5 (**E**), P6 (**F**) polymers at T = 70 °C. Point (a) determines the moment of addition an initiator−APS, point (b) the addition of the aqueous solution of the monomers−NIPA and appropriate PEGDMAs, point (c) the beginning of visible change in cloudiness of the reaction mixture, point (d) the complete turbidity of the reaction mixture.

Figure 2A–F show the time-dependent conductivity changes observed during the synthesis of P1–P6 at 70 °C. The conductivity change profiles among the P1–P6 mixtures demonstrate distinct differences during the first 20 s after the polymerization process began, as illustrated in the bottom plots of Figure 2A–F.

**Figure 3:**
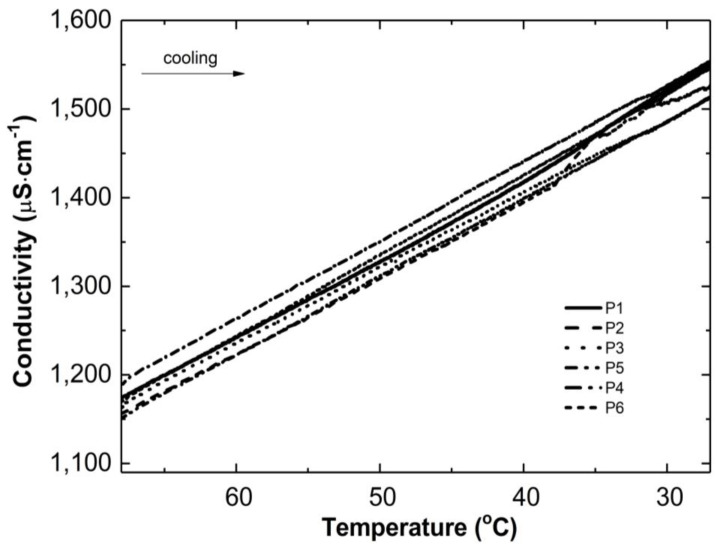
Conductivity changes versus temperature in the post-reaction mixtures of P1–P6 during the cooling process.

**Figure 4:**
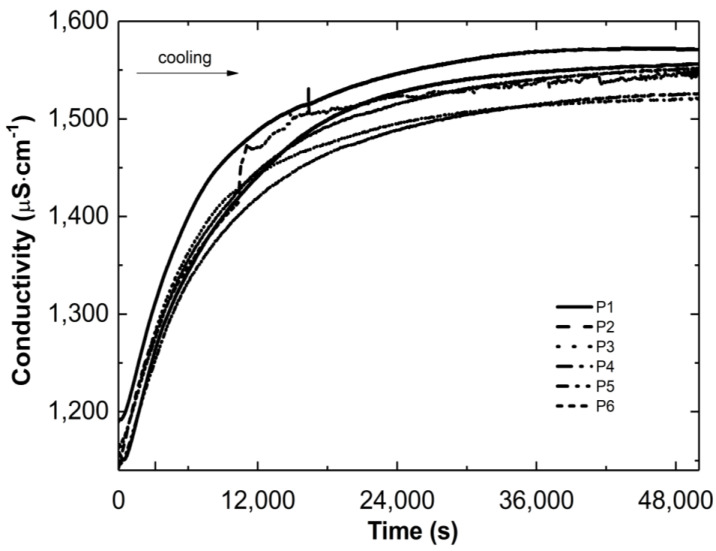
Conductivity changes versus time in the post-reaction mixtures of P1–P6 during the cooling process.

Figure 3 and Figure 4 show the conductivity profiles of the reaction mixtures P1–P6 as they cool to room temperature as a function of temperature and time, respectively.

### 3.3. Attenuated Total Reflection Fourier Transform Infrared Spectroscopy Analysis

**Figure 5:**
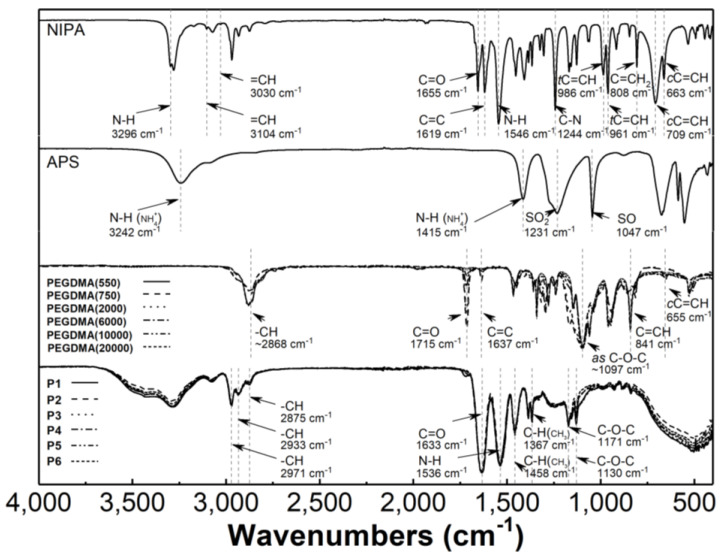
Fourier-transformed infrared spectroscopy with attenuated total reflectance (ATR−FTIR): spectra of monomer−N-isopropylacrylamide (NIPA), initiator−ammonium persulfate (APS), cross-linkers (PEGDMA), and synthesized polymers P1–P6.

The monomers NIPA and five PEGDMAs, the initiator APS, and the synthesized polymers P1–P6 were characterized using ATR-FTIR spectroscopy. Figure 5 displays the typical ATR-FTIR spectra of the substrates and products, with characteristic bands labelled. The spectra of NIPA and APS have been previously described in detail in our studies [52].

Peaks at approximately 1715, 1637, 1097, 841, and 655 cm^−1^ in the spectra of PEGDMA cross-linkers are attributed to C=O, C=C, C-O-C, C=C-H, and C=C-H bending vibrations, respectively [53,54,55,56,57].

The ATR-FTIR spectra of poly(NIPA-PEGDMA) P1–P6 exhibit peaks that characterize the C-H vibrations of the isopropyl group at 2971, 2934, and 2875 cm^−1^. Additionally, there is a peak attributed to the stretching vibrations of the CONH at 1636 cm^−1^, a peak corresponding to the N-H stretching vibrations at 1537 cm^−1^, and peaks originating from the C-O-C stretching vibration at 1171 cm^−1^ and 1130 cm^−1^ [58,59].

### 3.4. Hydrodynamic DiameterAnalysis 

**Figure 6:**
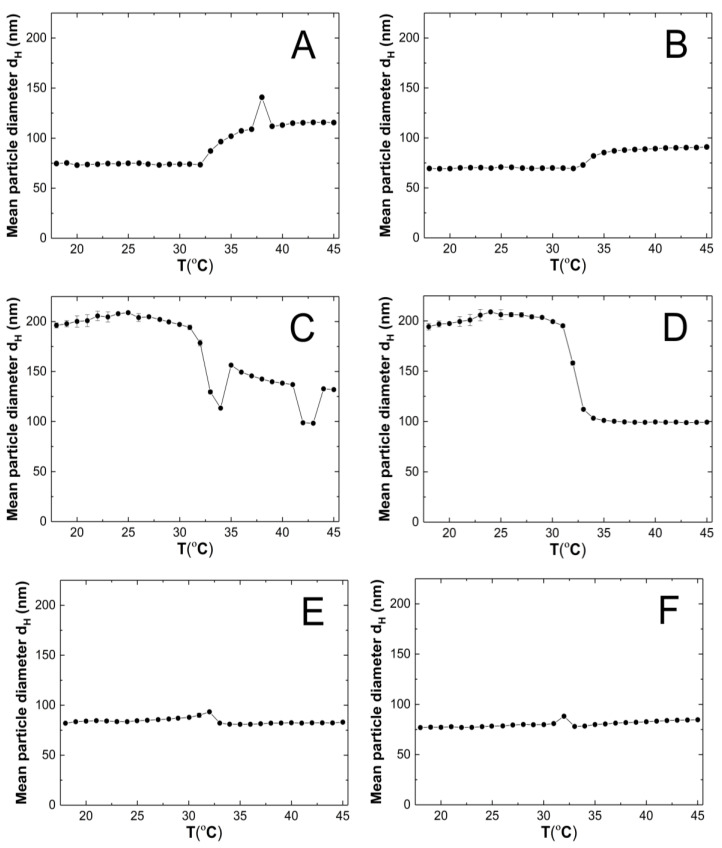
The changes in hydrodynamic diameter vs. temperature of the P1 (**A**), P2 (**B**), P3 (**C**), P4 (**D**), P5 (**E**), P6 (**F**) samples, determined by dynamic light scattering.

HD was measured at one-degree intervals between 18 °C and 45 °C. Five measurements were taken at each temperature, and the values were averaged with the standard error calculated. Figure 6A–F show the changes in the hydrodynamic diameter of purified aqueous dispersions of P1–P6 polymers with temperature.

**Figure 7:**
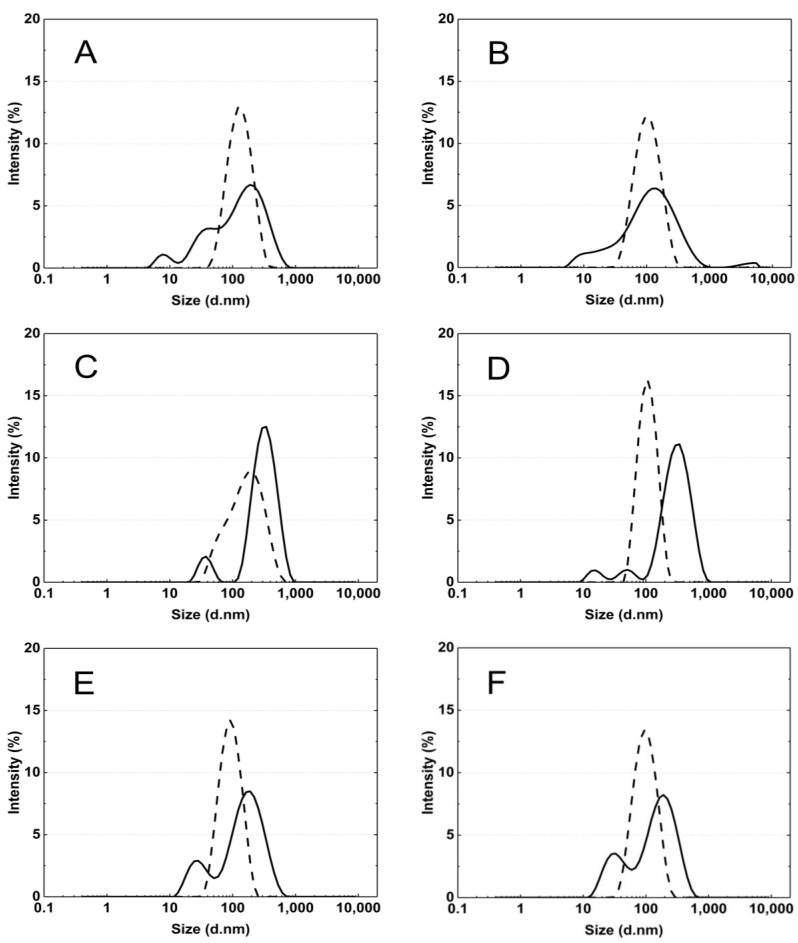
The particle size distribution by intensity for P1 (**A**), P2 (**B**), P3 (**C**), P4 (**D**), P5 (**E**), and P6 (**F**) dispersions at 18 °C—solid line and 45 °C—dash line, obtained from dynamic light scattering (DLS) analysis.

Figure 7A–F display typical intensity-based distribution plots of cross-linked polymer particles P1–P6 in an aqueous dispersion at 18 °C and 45 °C. The size distribution represents the most intense peak out of the five peaks recorded at a specified temperature.

### 3.5. Polydispersity Index

**Figure 8:**
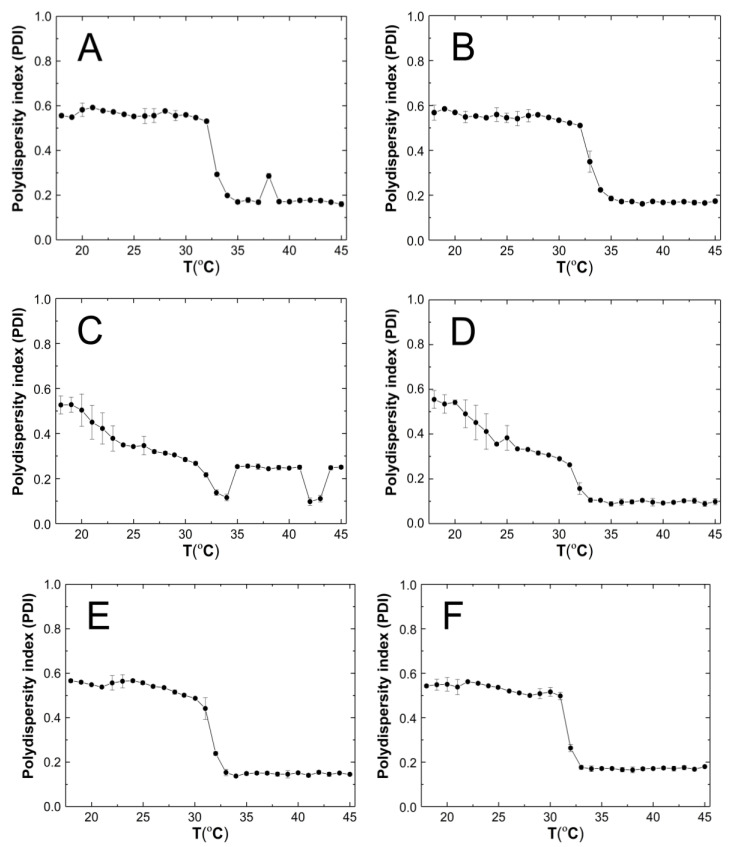
The influence of temperature on the polydispersity index (PDI) of P1 (**A**), P2 (**B**), P3 (**C**), P4 (**D**), P5 (**E**), and P6 (**F**) samples, determined by dynamic light scattering.

The polydispersity index (PDI) of the tested systems was measured during the particle size measurements. Five PDI measurements were taken at one temperature ranging from 18 to 45 °C. The PDI values at one temperature were averaged and the standard error calculated. Figure 8A–F show the variation of the PDI values for the purified aqueous dispersions of P1–P6 polymers as a function of temperature.

### 3.6. Zeta Potential

**Figure 9:**
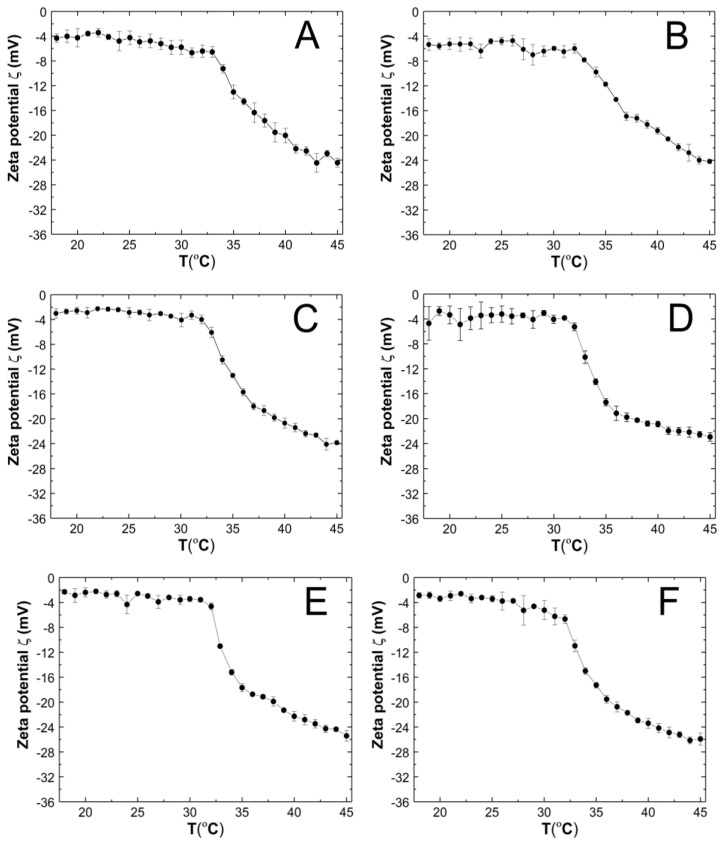
The influence of temperature on the zeta potential (ZP) of P1 (**A**), P2 (**B**), P3 (**C**), P4 (**D**), P5 (**E**), and P6 (**F**) samples, determined by electrophoretic mobility.

Zeta potentials (ZPs), also known as electrokinetic potentials, were measured for a purified aqueous dispersion of P1–P6 polymers over a temperature range of 18–45 °C. Five measurements were taken for each temperature, and the average value was calculated along with the corresponding standard error. Figure 9A–F illustrate the temperature-dependent changes in ZP. The samples were measured without buffering, and their pH values were measured at approximately 22.5 °C as follows: 6.2, 6.3, 5.7, 6.3, 6.3, and 6.7 for the P1, P2, P3, P4, P5, and P6 polymer dispersions, respectively.

The ZP values of all tested co-polymer dispersions were consistently negative throughout the temperature range of 18–45 °C. An increase in temperature above the phase transition temperature led to a linear decrease in ZP. At 45 °C, the ZP values were −24.44 ± 0.71, −24.18 ± 0.34, −23.86 ± 0.29, −22.94 ± 0.68, −25.42 ± 0.86, and −25.92 ± 0.97 mV for P1, P2, P3, P4, P5, and P6 respectively.

### 3.7. Thermogravimetric Analysis

**Figure 10:**
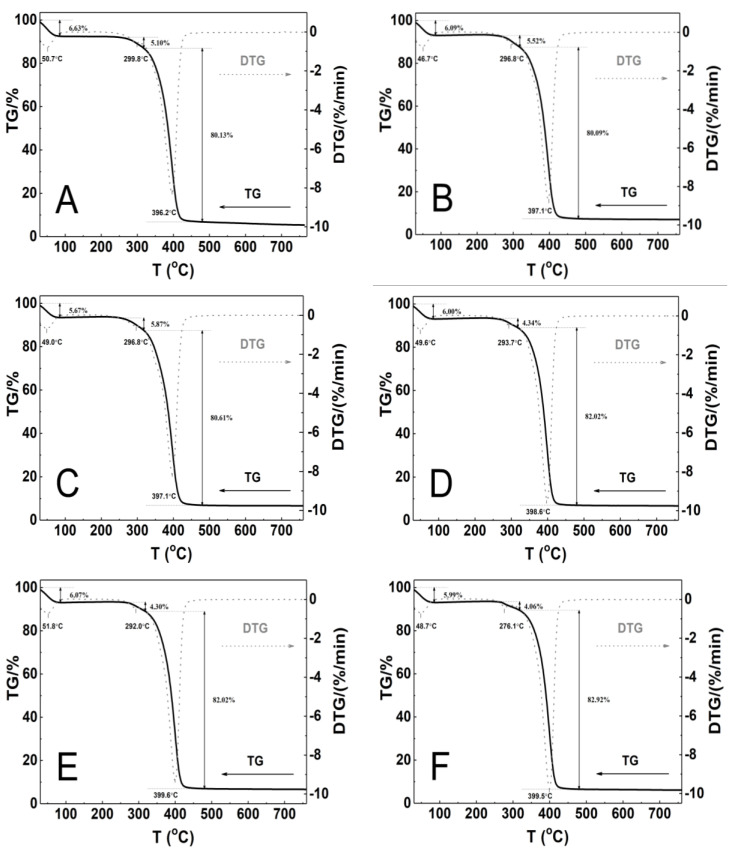
Thermoanalytical curves for polymers P1 (**A**), P2 (**B**), P3 (**C**), P4 (**D**), P5 (**E**), and P6 (**F**) obtained using a heating rate of β = 5 °C min^− 1^ in a nitrogen atmosphere at 50 mL/min. The curves include TG (solid line) and DTG (dashed line) data.

The polymeric material’s thermal stability was assessed by measuring mass changes between 25 and 800 °C. Figure 10A–F show the TGA and first derivative thermal analysis (DTA) plots, while Table 2 provides the TGA and DTA curve analysis results for all six polymers evaluated.

Thermographic plots show that all P1–P6 systems have similar three-stage mass loss profiles and thermal decomposition patterns. The initial stage of thermal degradation, from 30 to 85 °C, resulted in a mass loss ranging from 5.7% to 6.6%. A very small mass loss of 4.1% to 5.9% was observed between 249 and 318 °C. The greatest mass loss, approximately 81%, occurred in the temperature range 318–480 °C. The weight loss was minimal and gradual from temperatures above 480 °C until the final temperature. At the end of the process, the residual mass was 5.4% for P1 and 7.1% for P2, representing the lowest and highest values, respectively, compared to the initial mass. At 750 °C, the thermal decomposition of all polymers exceeded 90%, indicating complete polymer decomposition at this temperature. The DTG curves showed three peaks with maximum temperature ranges of 48.7–51.8 °C, 276.1–299.8 °C, and 396.2–399.6 °C. Table 2 presents the results of TG and DTG curve analysis for the six tested polymers.

### 3.8. Differential Scanning Calorimetry Analysis

**Figure 11:**
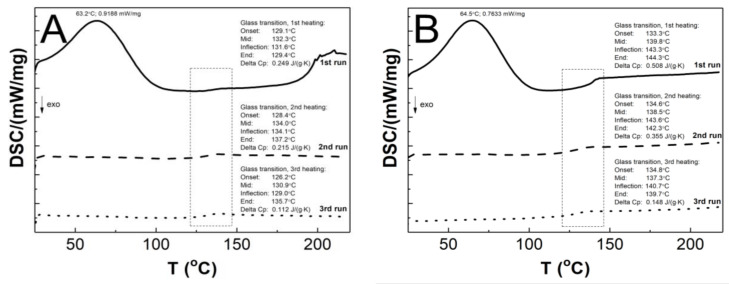

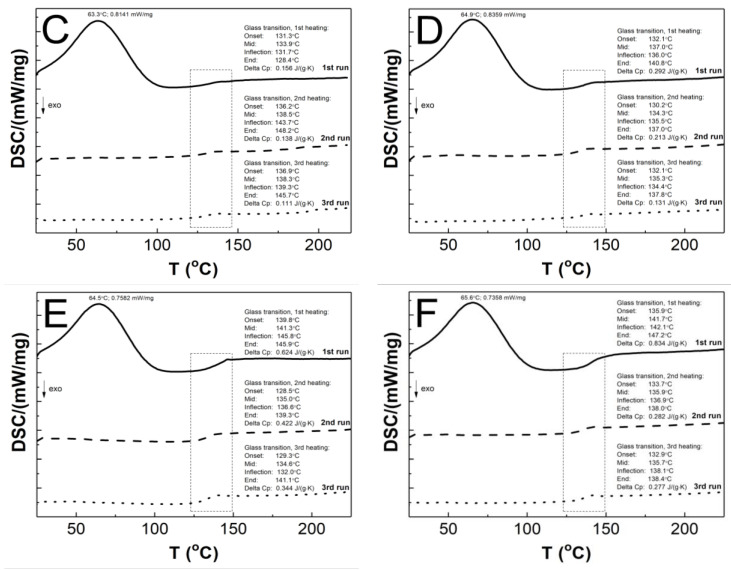
The DSC heating thermograms of the first (solid line), second (dashed line), and third (dotted line) heating runs for the polymers P1 (**A**), P2 (**B**), P3 (**C**), P4 (**D**), P5 (**E**), and P6 (**F**). The heating rate β = 5 °C min^−1^ in a nitrogen atmosphere at 50 mL·min^−1^.

Figure 11A–F present a comparative analysis of the thermal profiles of the first (solid line), second (dashed line), and third (dotted line) heating runs of the P1–P6 samples. All of the curves generated by the first heating cycle exhibit two endothermic effects, the broad endothermic peak at approximately 64.3 °C and a less pronounced endothermic event at around 137.7 °C. In the curves generated in the second and third stages, no endothermic peak was detected, and only one endothermic effect was visible. The glass transition temperatures T_g_ obtained from DSC studies of the first, second, and third run heat were 132.3/134.0/130.9; 139.8/138.5/137.3; 133.9/138.5/138.3; 137.0/134.3/135.3; 141.3/135.0/134.6; 141.7/135.9/135.7 °C, for the P1, P2, P3, P4, P5, and P6 respectively. The first, second, and third runs of heating yielded the following ∆Cp values: 0.249/0.215/0.112; 0.508/0.355/0.148; 0.156/0.138/0.111; 0.292/0.213/0.131; 0.624/0.422/0.344; 0.834/0.282/0.277 J·g^−1^·K^−1^ for P1, P2, P3, P4, P5, and P6, respectively.

### 3.9. Powder X-ray Diffraction Analysis

**Figure 12:**
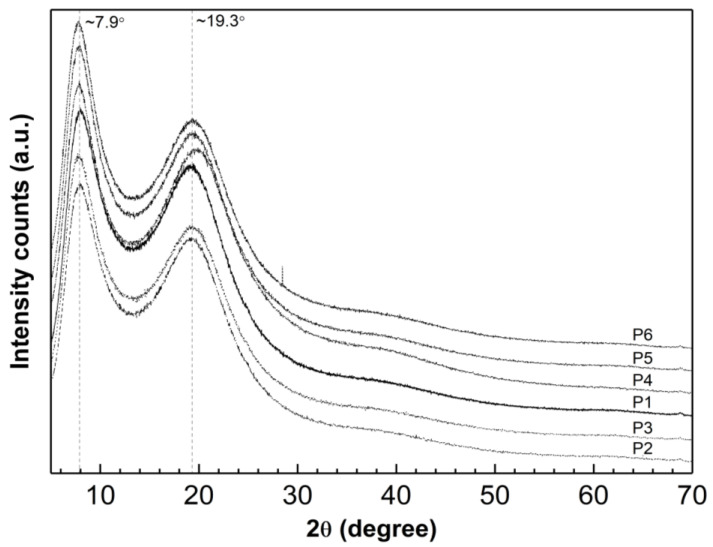
Powder X-ray diffraction patterns of synthesized polymers P1–P6; 2θ = 5°–70°.

Diffraction data were obtained for the lyophilized and powdered samples of synthesized P1–P6 polymers in the form of diffraction patterns. These patterns display the relationship between the intensity (count number) of diffraction reflections from diffraction angle (reflection Bragg) 2θ as presented in Figure 12. The PXRD patterns recorded show two wide diffraction pronounced peaks at 2θ values of approximately 7.90° and 19.30°.

## 4. Discussion

### 4.1. Synthesis

In order to obtain the polymeric particles of NIPA derivatives, the surfactant-free participation polymerization (SFPP) method was tested. Conducting the radical polymerization reaction at 70 °C in an aqueous medium without surfactant allows for easy observation of the process. The polymer’s thermosensitive properties caused the clear solution to turn into an increasingly milky biphasic mixture, indicating the progression of the polymer chain elongation process. The turbidity confirms that the obtained products are a thermosensitive polymer with a LCST. Above LCST, the polymer chain dehydrates and becomes insoluble [60,61,62]. The thermally controlled phase transition behavior is explained by thermodynamic changes in the system based on the Gibbs free energy equation [63].

There was no observed linear relationship between the length of the cross-linker chain and the time taken for turbidity to appear or for the plateau to be established. The conductivity in each system reached a constant level after approximately 23,000 s. The yield of the process increased as the length of the cross-linker chain increased (550–6000 Mn) for P1–P4 polymers. However, for P5–P6 polymers, the yield decreased. Increasing the molecular weight of the cross-linking agent above 6000 Mn, and thus lengthening its chain, may reduce the process yield. Turbidity decreased and finally disappeared as the reaction mixture cooled to room temperature.

### 4.2. Conductivity

Conductivity measurements were utilized to monitor the synthesis process in the reaction system and identify specific polymerization steps, consistent with our previous studies [52,64]. The changes in conductivity and temperature observed after adding APS to the reaction system were caused by the initiator’s decomposition, which generated free radicals (Figure 2A–F point (a)) [65]. The addition of the NIPA and PEGDMA mixture caused a notable reduction in conductivity (Figure 2A–F point (b)), indicating the initiation of the polymerization and cross-linking processes. This activation of the NIPA and PEGDMA molecules resulted in the formation of active compounds, specifically oligoradicals. The propagation of the polymer chain is reflected in the flattening of the graph of conductivity changes over time, which persisted for approximately one hour, during which the polymer chain elongated. The opacity of the reaction mixture (Figure 2A–F point (c)) is another visible indication of chain elongation and cross-linking reactions resulting from the mixture’s addition. This observation is consistent with the conductivity changes. The increase in conductivity, which began after approximately 8000 s, continued until the end of the process. This may indicate the presence of radical reactions that are not related to the elongation of the polymer chain structure, such as termination or radical center transfer, or other reactions that occur in an aqueous environment, such as hydrolysis or dissociation.

As the temperature of the reaction mixtures was lowered, an increase in the conductivity of the reaction system was observed over time and temperature (Figure 3 and Figure 4). The rising conductivity in the system may be attributed to the unwinding of polymeric strands and the release of ‘stuck’ compounds or functional groups from the coiled form, which dissociate after release. No significant deviations from the linear profile were observed, which could be indicative of the phase transition moment. The observed deviations from linearity in the P2 post-reaction system can be attributed to apparatus errors.

### 4.3. Attenuated Total Reflection Fourier Transform Infrared Spectroscopy

The analysis of the ATR-FTIR spectrograms presented in Figure 5 indicates that a pure compound, resulting from the complete polymerization of the substrates, was obtained, free from impurities. The dactyloscopic regions of the substrates and polymeric products are significantly different, further confirming the formation of a new product. The absorption bands present in the PNIPA derivatives spectra, which are characteristic of the bonding of C-O-C groups, indicate the success of the cross-linking process. The presence of a band characteristic of the hydroxyl group in the product’s spectrum indicates the existence of water in the tested PNIPA derivative samples. This observation confirms the presence of trace amounts of water that were not removed during the freeze-drying process.

### 4.4. Hydrodynamic Diameter

**Figure 13:**
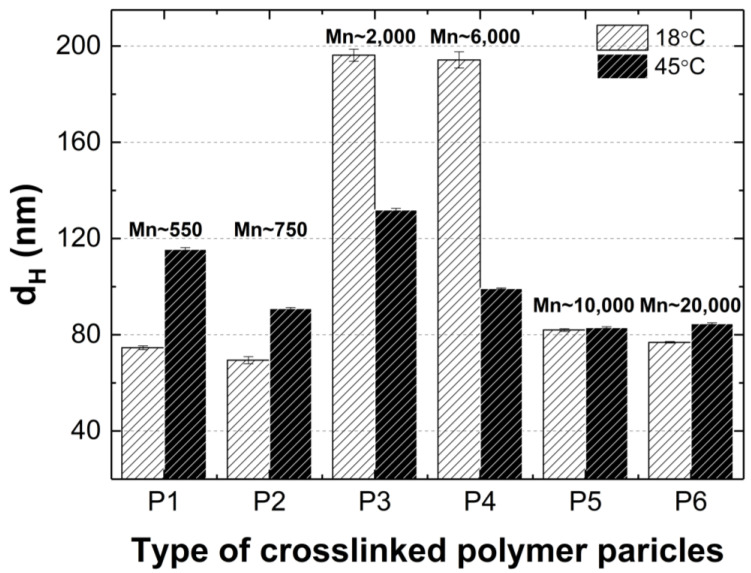
Hydrodynamic diameters of P1–P6 measured at 18 °C and 45 °C. The designations Mn 550, 750, 2000, 6000, 10,000, and 20,000 indicate the average particle size of the cross-linker in polymerization of P1, P2, P3, P4, P5, P6, respectively.

Figure 13 clearly shows that at 18 °C, the polymer particles P1, P2, P5, and P6 are nano-sized. Their (HD) does not exceed a width of 85 nm. In contrast, the HD of polymers P3 and P4 is almost 2.5 times larger. A correlation can be observed whereby polymers containing cross-linkers with the longest and shortest carbon chains in their polymer network have comparable hydrodynamic diameters at the nano scale. A similar relationship, although not as pronounced, is observed at 45 °C.

It is conceivable that the chain lengths of the cross-linking agents in polymers P3 and P4, in comparison to polymers P1, P2, P5, and P6, are optimal for allowing the polymer chains to be configured in such a way that in the temperature range of 18–32 °C, intramolecular hydrophobic interactions are intensified, resulting in intermolecular aggregation, uniformity in the size of the particles formed, and, ultimately, collapse of the polymer particles at the LCST [66].

It can be to assumed that the case in question is not affected by the polarity and size of particle solvent or its effect on the mobility of the polymer particles given that the same solvent was used in the same volume in each case. Nevertheless, the intermolecular dynamics during the polymerization kinetics, the architecture of the particles being formed, and the interaction of the polymer particles with each other and with the solvent particles can influence this situation. It can be observed that intermolecular interactions, particularly in relation to the formation of hydrogen bonds between polymer molecules and solvent molecules, appear to be of significant importance below the LCST in the context of polymers P3 and P4. This is due to the fact that at 45 °C, where the polymer–solvent hydrogen bonds are broken and intermolecular interactions increase, there is not such a significant difference in particle size among polymers P1–P6.

It seems probable that the steric configuration of the P3 and P4 polymer molecules within the dispersion system, the intrinsic structure of these polymers and the resulting pore size in the polymer network via cross-linking may facilitate the penetration of water molecules and the formation of hydrogen bonds within the polymer network. The formation of strong hydrogen bonds in which the proton donor is a water molecule results in the formation of ‘water shells’ and therefore maintains the original size of the particle. It is also important to consider the presence of NH_4_^+^ and SO_3_^−^ ions in the system, which result from the hydrolysis of terminal groups. It seems plausible to suggest that the pores formed in the polymer network are of a sufficient size to accommodate large sulphate ions originating from the terminal groups of the polymer chains. This may favor repulsive interactions and the formation of larger polymer particle sizes.

The observation that the particle size of the P1 and P2 polymers is larger at 45 °C than at 18 °C can be attributed to the larger tendency of aggregate formation after the LCST transition.

The particle size distribution analysis of the dispersion of P1–P6 polymers at 18 °C revealed the presence of at least three separated populations (cf. Figure 8), indicating that the system exhibits polydispersity at this temperature. It can be observed that as the cross-linking chain length increases, the particle size distribution becomes narrower, although it still remains polymodal, a finding that is consistent with the PDI measurement results. It may be hypothesized that systems at temperatures below the LCST display a lack of stability due to the constant state of competition between polymer molecules for the formation of hydrogen bonds and the attainment of equilibrium. The particle size distribution at 45 °C appears to be monomodal and narrower in comparison to the size distribution at 18 °C. The data suggest that the polymers underwent a reduction in size by shrinking, resulting in a decrease in HD for polymers P3–P6. Conversely, the HD of polymers P1 and P2 exhibited an increase, which may be indicative of particle aggregation forming stable aggregates—mesoglobules [67]. This can be related to the lifetime of the hydrogen bonds and also to the volume of the solvation shell. It is therefore to be expected that this will automatically affect the entropy of hydration, which contributes to the free energy of the system [68,69]. Above the phase transition temperature, coil globules are no longer stabilized by the surrounding solvent. Consequently, the tendency to aggregate and form mesoglobules is observed in the case of P1 and P2. However, in the case of P3-P6, it can be hypothesized that the longer cross-linker chain may provide stabilization and protection against aggregation [70,71,72].

### 4.5. Polydispersity Index

As anticipated, the PDI of the P1–P6 polymeric derivatives of PNIPA exhibited a notable variation in particle size at the phase transition temperature, as illustrated in Figure 9A–F. At temperatures below the phase transition temperature, the PDI values did not exceed 0.6. This indicates that within the temperature range of 18–32 °C, the samples exhibited a relatively high polydisperse character. A PDI value exceeding 0.7 is considered excessively polydisperse, rendering the sample unsuitable for particle distribution analysis [73]. PDI values below the phase transition temperature were observed to be in the range of 0.2–0.4, indicating that the size of the dispersed P1–P6 polymer particles gradually became uniform under thermal energy, resulting in monodispersity [52,74]. The observed decrease in PDI values within the temperature range of 18–32 °C for samples P3 and P4 may be attributed to discrepancies between experimental outcomes and existing theoretical frameworks that describe systems comprising spherical particles. The Einstein–Stokes relation assumes a hydrodynamic radius for spherical particles. However, the polymer dispersions under investigation may contain particles that exhibit either spherical or non-spherical shapes. This may result in varied chain density distributions due to reduced solvent interactions and also due to Brownian fluctuations, which may affect the divergence of results. It should also be emphasized again that measurements made using the DLS method give the hydrodynamic diameter and not the actual particle size [75].

### 4.6. Zeta Potential

One of the principal factors influencing the interaction of particles in a colloidal system is their charge [76,77]. In order to determine the dimensions of the polymeric particles, it is also necessary to ascertain their surface charge in order to ensure accurate characterization. According to theory proposed by Derjaguin, Landau, Verwey, and Overbeek (DLVO), the stability of the colloid depends on the sum of the attractive van der Waals forces and the electrostatic repulsion forces resulting from the presence of the electrical double layers of the two uniquely charged surfaces. Thus, the zeta potential value provides an estimate of the stability of the colloidal system. Furthermore, the zeta potential value can be employed to indicate the combination of particles with opposite zeta potential values, which represents a key factor in the design of carrier–drug combinations

The resulting polymer particles P1–P6 are negatively charged throughout the temperature range 18–45 °C (Figure 10A–F). Their zeta potential values range from approximately −2.0 mV to −26 mV. According to the principles of zeta potential theory, the occurrence of high potential values (either negative or positive) with a magnitude greater than 30 mV is an indication of suspension stability [78]. It is important to note that the zeta potential, as an absolute value, provides information on electrostatic repulsive forces but not on attractive van der Waals forces. Consequently, it is not uncommon to find stable solutions of particles characterized by low zeta potential values and vice versa. Therefore, if the van der Waals attractive forces become weak, it is possible that mild electrostatic repulsion, as described by low zeta potential values, may be sufficient to ensure the stability of the colloid [79,80].

The observed increase in charge density on the surface of the particle with increasing temperature may be attributed to the phenomenon of shrinkage. During this process, relatively large sulphonic groups are pushed out of the polymer network and onto the surface of the particle. This results in an increase in charge density. In a system comprising both negatively and positively charged segments, electrostatic attraction between them can result in heteroaggregation or agglomeration [81]. It is probable that this phenomenon occurred in systems P1 and P2, as evidenced by an increase in particle size above the phase transition temperature rather than a decrease.

The measurements exhibited minimal standard errors, which may suggest that the incorporation of PEGDMA cross-linkers could play a stabilizing role in the systems tested by forming a steric barrier, but they also reduced the zeta potential [76,82].

The results of the zeta potential measurements did not allow for a clear linear relationship between the length of the cross-linking chain and the zeta potential value to be established. Nevertheless, it was observed that the zeta potential values at 45 °C were comparable for pairs of cross-link-containing polymers of comparable orders of magnitude in terms of chain length. For instance, polymers P1 and P2, P3 and P4, and P4 and P5 exhibited comparable zeta potential values at 45 °C. However, no such relationship was observed at 18 °C. This phenomenon can be attributed to the specific conformation of the long-chain structure of the cross-linking agent and its coverage of the particle surface during the shrinkage of the polymer particles at high temperatures. Furthermore, it is possible that the specific adsorption of dissolved ions onto the formed, shrunken surface may also be a contributing factor. In both instances, this results in a reduction in the electrochemical thickness of the double layer. This results in a reduction in the maximum energy barrier, which in turn leads to particle aggregation [79,80,83,84].

### 4.7. Thermogravimetry

The TG/DTG curves for P1–P6 polymers heated in a controlled nitrogen atmosphere from 25 to 800 °C at heating rate 5 °C·min^−1^ are presented in Figure 11A–F. The weight loss runs exhibit a high degree of similarity, with three distinct stages discernible. The convergence of the thermal decomposition profiles indicates that the degradation mechanism of the tested P1–P6 polymers is the same. This is in line with the assumption that the polymers are made from the same substrates.

The initial stage observed below 85 °C may be attributed to various factors, including the removal of physically adsorbed water on the surface of the polymer particles and volatile substances or structural dehydration (~6.1%) [52,85,86,87,88,89]. The slight weight loss observed in the second stage, estimated at approximately 4.9%, can be attributed to the commencement of polymeric matrix decomposition and the release of ammonia and sulfur dioxide as a consequence of the decomposition of the terminal ammonium sulphate groups derived from the initiator [52,90]. The third main stage, characterized by the fastest rate of weight loss and the greatest percentage weight loss (~81%), can be attributed to the polymer chain backbone [91,92].

The onset decomposition temperature (*T_onset_*) of polymers P3 and P4 is significantly higher than for polymers P1, P2, P5, and P6 (cf. Table 2). This is likely due to the larger particle size of P3 and P4, which is consistent with HD measurements (cf. Figure 7A–F and Figure 13). The results do not align with the initial assumptions. It was anticipated that the elongation of the cross-linker chain would result in an increase in the *T_onset_* value, thereby enhancing the stability of the material. All polymers that were subjected to testing demonstrated stability up to 240 °C, see Figure 11A–F.

The percentage of residue remaining after 750 °C is remarkably consistent across all polymers tested, with an average of 6.4 ± 0.6. However, it is notable that the lowest residue is observed for P1 and the highest for P2 (cf. Table 2). This may indicate that the decomposition process of P1–P6 polymers occurred in a uniform manner across all samples and was not influenced by factors such as the presence of crystalline structures, as corroborated by PXRD studies (cf. Figure 13), or impurities [93,94,95].

The maximum degradation rate temperature for P1–P6 polymers exhibits near-identical values, with a standard deviation of ± 1.4 °C. This finding suggests that the effect of cross-linker chain length on degradation temperature is both negligible and non-linear.

### 4.8. Differential Scanning Calorimetry

As illustrated in Figure 11A–F, the endothermic effect observed at approximately 64 °C only manifests in the curve representing the first heating cycle. It is very likely that this phenomenon is related to dehydration, which can be involved as the evaporation of either hygroscopic water or poorly bound water from the polymer network [91]. This observation can be corroborated with the result obtained in TG measurements, cf. Figure 11. The results of the DSC studies, from second and third heating cycles, indicated that all polymers tested exhibited a single endothermic effect related to the glass transition phenomenon. Furthermore, no melting effects were observed. These findings imply that the samples under investigation were purely amorphous polymers, devoid of impurities or eutectic forms. Contaminated samples, on the other hand, exhibit multiple peaks, with the eutectic peaks becoming less distinct, while the amorphous polymers display no melting effect [96,97,98]. The Tg values obtained in the study of P1–P6 polymers are comparable within the acceptable experimental range of ±2.01 standard error, indicating that there is no linear effect of cross-linker chain length on this parameter. However, when comparing the heat flow values for the endothermic peak from the first heating run, it can be observed that the heat flow values for P3 and P4 are higher than for the other polymers. This heat flow difference may be attributed to the particle size, as evidenced by the HD study. This observation may indicate that particle size is influenced by factors beyond the conformations of the polymer chain and the strength of inter- and intramolecular interactions. It may also be affected by the geometry of the pores present in the parent polymer matrix filled with water molecules.

### 4.9. Powder X-ray Diffraction

X-ray diffraction studies indicate that the P1–P6 polymerization products contain a significant amount of the amorphous phase. This is demonstrated by the absence of sharp peaks that are characteristic of the periodicity of the crystalline form, as shown in Figure 13. This observation suggests that the substrates reacted completely and that there are no impurities with a crystalline structure in the final polymerization product. However, it has been reported that broad diffraction lines in nano-sized materials may indicate low crystallinity [99,100].

The tested samples showed a halo with two maxima at approximately ~7.90° and ~19.30° 2θ. In the PXRD pattern for P5, only the second maximum is shifted by 0.50° towards higher angles. This suggests that a change has occurred in the local molecular packing arrangement and indicates that the length of the cross-linker chain does not significantly affect the peak position [101,102].

Analysis of the peak shape and intensity in the diffractograms indicates that the structures of the tested substances are very similar. No new peaks appear, nor do any characteristic peaks disappear.

According to the PXRD data presented in our previous publication, the diffraction patterns of pure monomer—NIPA—and initiator—APS—show sharp and intense crystalline peaks [52].

## 5. Conclusions

In conclusion, the synthesis of six thermosensitive polymeric derivatives of NIPA (P1–P6) cross-linked by PEGDMA with different chain lengths (Mn 550–20,000) was successfully achieved via surfactant-free precipitation polymerization in an aqueous environment at 70 °C. The analysis of ATR-FTIR spectrograms demonstrated that the compounds obtained are the result of complete polymerization of the substrate, with no evidence of impurities. The deployment of conductivity measurements throughout the synthesis process can prove invaluable for the estimation of polymerization steps and their corresponding durations. Furthermore, it can serve as substrate for the study of reaction kinetics. The effect of the PEGDMA chain length on the physicochemical properties of the resulting co-polymers was examined. The synthesized polymers P1, P2, P5, and P6 were nano-sized, with an HD of less than 100 nm at 18 °C. The polymers P3 and P5 exhibited micro-sized characteristics, with an HD of approximately 195 nm at 18 °C. The synthesized polymers exhibited LCST within the range of 31 to 32 °C, which is in close proximity to the temperature of the human body at the surface. The behavior of the LCST was monitored by measuring changes in HD over the temperature range from 18 to 45 °C using the DLS method, with the results being confirmed by independent ZP measurements over the same temperature range. A linear relationship was not observed between the increase in PEGDMA chain length in the P1–P6 polymers and their HD and LCST values. The particle size distributions exhibited a constant and narrow distribution at 45 °C. The PDI values demonstrated a reduction in polydispersity with an increase in temperature above the LCST. The ZP results indicate the formation of particles with a negative surface charge within the temperature range of 18–45 °C. Furthermore, there is a tendency for colloidal stability to increase with increasing temperature once the LCST is exceeded. Nevertheless, the potential values remain within the range characteristic of unstable systems, with values between −30 and 30 mV.

No linear correlation was observed between the changes in thermogravimetric parameters and the length of the cross-linker chain in TG studies. The relative thermal stability was evaluated up to 240 °C. The DSC studies confirmed the absence of a linear relationship between glass transition temperature and cross-linker chain length. As evidenced by PXRD studies, a material exhibiting an amorphous halo was obtained, which may indicate the presence of multiple amorphous phases or disordered states, such as monocrystalline disorder. The physicochemical characteristics of the synthesized P1–P6 products can be employed to optimize the technological process of particles with the desired functional properties and to determine the suitability of macromolecular combinations for pharmaceutical applications. 

## Figures and Tables

**Table 1:** Substrate compositions of P1, P2, P3, P4, P5, and P6 cross-linked polymers.

Components		Type of Co-Polymer Nanoparticle System					
		P1	P2	P3	P4	P5	P6
Monomer (g)	NIPA	5.0099	4.9911	5.0246	5.0090	5.0022	5.0020
Anionic initiator (g)	APS	0.5051	0.5062	0.5037	0.5054	0.5017	0.5005
Cross-linkers (g)	PEGDMA (Mn~550)	0.5164	-	-	-	-	
	PEGDMA (Mn~750)	-	0.5383	-	-	-	
	PEGDMA (Mn~2000)	-	-	0.5080	-	-	
	PEGDMA (Mn~6000)	-	-		0.5088	-	
	PEGDMA (Mn~10000)					0.5041	
	PEGDMA (Mn~20000)	-	-				0.5005

**Table 2:** The results of the TG and DTG curve analysis of P1–P6.

Type of Polymer Nanoparticle System	t_1_ (°C)	Rate of Mass Loss 1 (% min^−1^)	t_2_ (°c)	Rate of Mass Loss 2 (% min^−1^)	t_3_ (°C)	Rate of Mass Loss 3 (% min^−1^)	*T*_Onset_ (°C)	*T*_Endset_ (°C)	Res. at 750 °C (%)	*T*_1.0wt%_ (°C)
P1	50.7	0.80	299.8	0.59	396.2	8.29	313.3	417.1	5.39	30.8
P2	46.7	0.75	296.8	0.65	397.1	8.67	312.2	416.2	7.06	30.8
P3	49.0	0.68	296.8	0.58	397.1	8.16	319.6	417.6	6.63	30.9
P4	49.6	0.71	293.7	0.47	398.6	9.49	327.8	414.2	6.71	30.6
P5	51.8	0.72	292.0	0.47	399.6	9.34	316.3	412.8	6.65	30.6
P6	48.7	0.72	276.1	0.44	399.5	9.76	318.1	412.9	6.12	30.2

## Data Availability

The original contributions presented in the study are included in the article, further inquiries can be directed to the corresponding author.
